# A phase II open label trial evaluating safety and efficacy of a telomerase peptide vaccination in patients with advanced hepatocellular carcinoma

**DOI:** 10.1186/1471-2407-10-209

**Published:** 2010-05-17

**Authors:** Tim F Greten, Alejandro Forner, Firouzeh Korangy, Gisele N'Kontchou, Nathalie Barget, Carmen Ayuso, Lars A Ormandy, Michael P Manns, Michel Beaugrand, Jordi Bruix

**Affiliations:** 1 Department of Gastroenterology, Hepatology and Endocrinology, Hannover Medical School, Carl Neuberg Strasse 1, 30625 Hannover, Germany; 2 Twincore Center for Experimental and Clinical Infection Research, Feodor-Lynen Strasse 7-9, 30625 Hannover, Germany; 3 Service d'Hepato-Gastroenterologie, Hopital Jean Verdier AP-HP, Universite' Paris XIII, Bondy 93143, Cedex, France; 4 BCLC Group, Liver Unit, Hospital Clínic. University of Barcelona. IDIBAPS. CIBEREHD. Villarroel 170, Barcelona 08036, Spain; 5 Department of Legal Medicine, University Medical Center Göttingen, Robert-Koch-Straße 40, 37075 Göttingen, Germany; 6 Medical Oncology Branch, National Cancer Institute, NIH, Bldg. 10 Rm 12N226, 9000 Rockville Pike, Bethesda MD 20892, USA

## Abstract

**Background:**

The sole effective option for patients with advanced HCC is sorafenib and there is an urgent need to develop new therapeutic approaches. Immunotherapy is a promising option that deserves major investigation. In this open label, single arm clinical trial, we analyzed the effect of a low dose cyclophosphamide treatment in combination with a telomerase peptide (GV1001) vaccination in patients with advanced HCC.

**Methods:**

40 patients with advanced HCC were treated with 300 mg/m^2 ^cyclophosphamide on day -3 followed by GM-CSF + GV1001 vaccinations on days 1, 3, 5, 8, 15, 22, 36 followed by 4-weekly injections. Primary endpoint of this phase II trial was tumor response; secondary endpoints evaluated were TTP, TTSP, PFS, OS, safety and immune responses.

**Results:**

None of the patients had a complete or partial response to treatment, 17 patients (45.9%) demonstrated a stable disease six months after initiation of treatment. The median TTP was 57.0 days; the median TTSP was estimated to be 358.0 days. Cyclophosphamide, GV1001 and GM-CSF treatment were well tolerated and most adverse events, which were of grade 1 or 2, were generally related to the injection procedure and injection site reactions. GV1001 treatment resulted in a decrease in CD4^+^CD25^+^Foxp3^+ ^regulatory T cells; however, no GV1001 specific immune responses were detected after vaccination.

**Conclusions:**

Low dose cyclophosphamide treatment followed by GV1001 vaccinations did not show antitumor efficacy as per tumor response and time to progression. Further studies are needed to analyze the effect of a combined chemo-immunotherapy to treat patients with HCC.

**Trial registration:**

NCT00444782

## Background

Hepatocellular carcinoma (HCC) represents the third leading cause of cancer related death worldwide. Only a minority of patients is eligible for potential curative treatments such as surgical resection, liver transplantation and local ablative therapies [1,2]. Therapeutic options for patients with advanced HCC are limited. So far, Sorafenib, a multi-targeted tyrosine kinase inhibitor, is the only drug, which leads to an increase in overall survival in patients with advanced HCC as demonstrated in two randomized phase III placebo-controlled trials [3,4]. Sorafenib delays tumor progression but as it does not achieve tumor resolution and tumor free long term survival there is major need to develop new options that would further increase the current therapeutic benefits, Therefore, new treatment options are urgently needed for patients with HCC at several phases of their evolution and recommendations regarding the optimal trial designs have been published [2].

Immunotherapy represents a potentially attractive option for HCC patients [5]. Cancer vaccines using peptides derived from tumor-specific antigens represent one potential alternative immunotherapeutic procedure. A number of different tumor antigens identified in HCC represent potential antigens for a peptide-based vaccination approach in patients with HCC [5]. Telomerase activity has been expressed in numerous tumors including HCC [6,7] and immunogenic telomerase peptides have been characterized [6]. Recently, it has also been reported that telomerase-specific CD4^+ ^and CD8^+ ^T cell responses are induced upon vaccination with hTERT-transfected dendritic cells [8] and vaccination with telomerase derived peptide, GV1001, was shown to induce T cell responses in patients with non-resectable pancreatic cancer and non-small cell lung cancer [9,10].

However, the effect of a cancer vaccine might be inhibited by the presence of CD4^+^CD25^+ ^regulatory T cells [11], which are known to suppress the function of antigen-specific T cell responses and are increased in patients with HCC [12]. Previously, we have been able to demonstrate that low dose cyclophosphamide treatment can impair the effect of regulatory T cells in patients with HCC [13]. Based on these studies we have now investigated the effect of a telomerase peptide (GV1001) cancer vaccine in combination with a low dose cyclophosphamide treatment.

## Methods

### Patient population

The study population consisted of male or female patients (≥ 18 years of age) with advanced-stage hepatocellular carcinoma, which was either confirmed histologically or diagnosed according to European Association for the Study of the Liver criteria of known predisposing chronic liver disease, alpha-fetoprotein (AFP) > 400 ng/mL, and characteristic imaging. Patients were classified as having advanced disease if they were not eligible for or had disease progression after surgical or locoregional therapies. The eligibility criteria also included an Eastern Cooperative Oncology Group (ECOG) performance status score of 1 or less, Child-Pugh liver function class A, a life expectancy of 12 weeks or more, adequate hematologic function (platelet count, ≥ 75-10^9 ^per liter; hemoglobin, ≥ 9.0 g per deciliter; white blood cell count ≥ 3.0 × 10^9 ^per liter), total bilirubin, ≤ 2 mg per deciliter [51.3 μmol per liter]; alanine aminotransferase and aspartate aminotransferase, ≤ 5 times the upper limit of the normal range) and adequate renal function (serum creatinine, ≤ 1.5 mg per deciliter [132 μmol per liter]). Patients were required to have at least one untreated target lesion that could be measured in one dimension, according to the Response Evaluation Criteria in Solid Tumors (RECIST). Patients with known co-existing autoimmune diseases or HIV infection were excluded from the study. Patients were not eligible if they had received any type of an anti-tumor treatment or corticosteroids within the 4 weeks of pre-treatment with cyclophosphamide. All patients provided written informed consent before enrollment in the study. The study was approved by the institutional review board or ethics committee at each center and complied with the provisions of the Good Clinical Practice guidelines and the Declaration of Helsinki and local laws. The trial has been registered in clinicaltrials.gov database (NCT00444782). At the time of trial initiation the benefits of sorafenib had not been established. Thus only patients ineligible for therapies with higher priority (resection, transplantation, ablation and chemoembolization) were enrolled as well as patients, who had progressed under those treatment types. Upon demonstration of the benefits of sorafenib, the patients ineligible for sorafenib therapy due to medical reasons wee also included.

### Treatment Plan

Patients received intravenous infusion of 300 mg/m^2 ^cyclophosphamide on day -3 followed by intradermal immunizations with 0.56 mg GV1001 and 75 μg granulocyte macrophage colony-stimulating-factor (GM-CSF) on days 1, 3, 5, 8, 15, 22, 36 followed by 4-weekly injections. Adverse events/toxicities were categorized and graded by the Common Terminology Criteria of Adverse Events (version 3.0). There was no dose modification. Criteria for discontinuation included unacceptable toxicities and symptomatic disease progression defined as a 2-step increase in the patient's performance status (to be confirmed after 2 weeks). Additionally, treatments were stopped for serious concurrent illness or significant worsening of concurrent illness.

### Delayed type hypersensitivity (DTH) analysis

DTH skin reaction (size of skin reaction) was examined 48 hours after administration. The DTH-test was considered positive if the area of the skin reaction had an average diameter of > 5 mm at 48 hours after administration. DTH tests were performed on days 1, 8, 15, 22, 36, and at week 10.

### In vitro analysis of immune responses

Peripheral blood mononuclear cells (PBMC) were obtained from patients by Ficoll density gradient centrifugation (Biochrom, Berlin, Germany) at the indicated time points as previously described [14]. The following fluorochrome-labled anti-human antibodies were used in this study to detect regulatory T cells: anti-CD3 (clone UCHT1; eBioscience), anti-CD4 (clone RPA-T4, BD Pharmingen), anti-CD25 (clone 4E3; Miltenyi Biotec), anti-Foxp3 (clone 206D; Biolegend). Stained cells were washed and analyzed with a FACSCalibur (BD Biosciences, Heidelberg, Germany) flow cytometer. All data analysis was performed with CellQuest software (BD Biosciences, Heidelberg, Germany). Isotype-matched antibodies were used as controls. For analysis of antigen-specific T cell responses 5 × 10^5 ^PBMC were plated in 96 well plates and 1 μM peptide was added. IFN-γ was measured in supernatants after 24 hours by ELISA and proliferation was measured by H-3 thymidine incorporation after 72 hours. Antigen-specific T cell responses were analyzed before treatment and 3, 6 and 12 weeks after first peptide vaccination.

### Statistical analysis

The primary endpoint of this study was response rate according to RECIST after 6 months [2]. All eligible patients who began treatment were considered assessable for the primary end point. The primary efficacy endpoint, the response rate (CR+PR) according to modified RECIST, was based on the Best Overall Response and was to be presented using frequency count and percentage. The number of patients in the considered analysis population was used as denominator for calculation of the rate. The 95% confidence interval around the response rate was also to be presented. The analysis of the primary endpoint was to be presented for the Intent-to-Treat (ITT) population. Secondary end points included TTP, time to symptomatic progression (TTSP), PFS, toxicity profile and immune responses. TTSP was defined as the time from the date of the first administration of GV1001 to the date of symptomatic progression (a 2-step deterioration in performance status) or death. The change in performance status was confirmed after 2 weeks. Subjects who had no documented symptomatic progression at the end of the study or who were lost to follow-up prior to having symptomatic progression were censored at the date of their last visit or contact. OS was not a pre-defined Endpoint. Kaplan-Meier methodology was used to describe the distribution of TTP, PFS, TTSP and survival. All analyses were performed using SAS version 8.1 or later (http://www.sas.com; SAS Institute, Cary, NC).

## Results

### Characteristics of patients

40 patients with advanced HCC (BCLC-C) were enrolled between November 2006 and April 2008 at three different European centers. 14 patients were enrolled in Spain, 12 patients in Germany and 14 patients in France. Alcoholic liver cirrhosis was the predominant cause of liver disease (40%) and 95% of the patients had a Child-Pugh A liver cirrhosis. 26 patients (65%) had a BCLC tumor stage of C. A summary of the baseline characteristics of all patients is presented in Table 1. All patients were eligible for safety and efficacy analysis, with the exception of three patients, which were not assessable due to clinical progression or death before the treatment was initiated.

**Table 1:** Demographic and baseline characteristics:

**Demographics**	
Age Mean	66.5 ± 10.5
Sex (male/female)	35/5
White/Caucasian	36 (90%)
	
**Cause of disease**	
HBV/HCV/Ethanol	5 (12.5%)/15 (37.5%)/16 (40%)
	
**ECOG - no (%)**	
0	31 (77.5%)
1	9 (22.5%)
	
**BCLC stage - no (%)**	
A	2 (5%)
B	12 (30%)
C	26 (65%)
	
**Child - Pugh Score - no (%)**	
A	38 (95%)
B	2 (5%)
	
**Prior Therapies - no (%)**	
Prior therapy received	26 (65%)
Surgery	9 (23%)
Other type of therapy	25 (63%)
	
**Biochemistry profile - Median ± SD (range)**	
ALT (IU/L)	57.8 ± 31.1 (range 16 - 166)
AST (IU/L)	81.2 ± 49.8 (range 23 - 229)
Bilirubin (μmol/L)	17. 7 ± 12.8 (range 5.5 - 54.5)

### Clinical trial conduct

According to the protocol, the recruitment of patients was stopped after the first interim analysis once 40 patients had been enrolled in this trial. At this time point treatment was stopped in 8 patients prior to three months of treatment, in 16 patients between months 3 and 6, in 7 patients between months 6 and 9 and in 7 patients between months 9 and 12. Two patients received treatment for more than 12 months. Imaging studies (CT or MRI) were performed before start of treatment and every 8 weeks thereafter. However, no objective tumor responses were detected in any of the patients after 6 months of treatment. All patients were evaluable for safety analysis and efficacy evaluation.

### Safety events

Patients received a median of 11.0 doses of GV1001 (range 6.0 to 19.0 doses). Sixteen patients (40.0%) received treatment for a minimum of 6 months. The overall incidence of treatment-related adverse events was 82.5%. Adverse events related to GV1001, GM-CSF or cyclophosphamide treatment occurred in 52.5%, 52.5% and 7.5% respectively. Most reported adverse events were related to the injection procedure and injection site reactions. The majority of adverse events related to GV1001 or GM-CSF were predominantly Grade 1, with a few Grade 2 events. A similar toxicity profile was observed for GM-CSF and only 4 adverse events were related to the pre-treatment with cyclophosphamide. Except for one case of renal failure (Grade 3), they were all grade 1 or 2 (Table 2).

**Table 2:** Incidence of drug-related adverse events

**Adverse event**	Grade 1/2 (*)	**3 (**†**)**
Overall incidence	33 (any grade)	
Injection site conditions	9/0	0
Pyrexia	2/1	0
Erythema	5/0	0
Renal failure	0/0	1

* Only side effects, which have occured in more than 5% of the patients are listed

† Any grade 3 adverse event has been listed

### Efficacy

**Figure 1:**
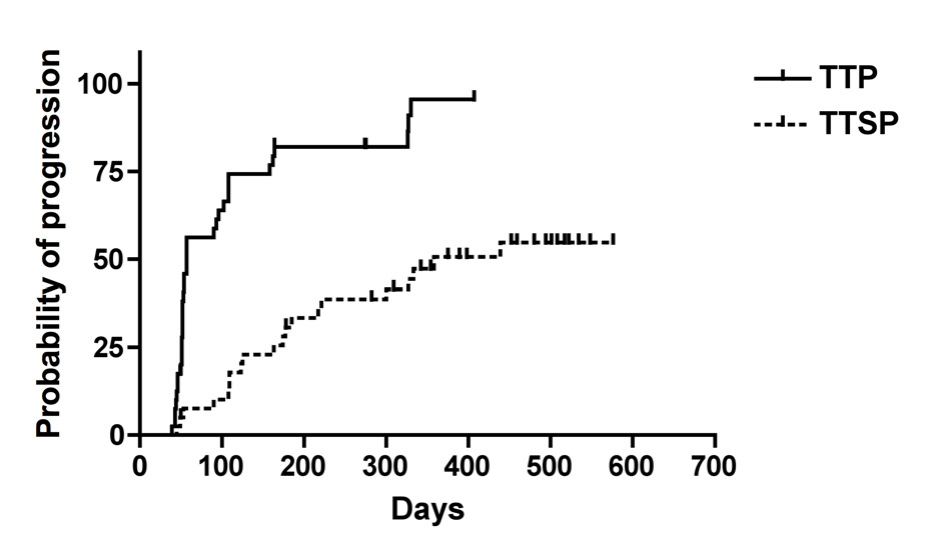
**Kaplan-Meier Analysis of Time to Progression (TTP) and Time to Symptomatic Progression (TTSP)**. The median TTP was 57 days (1.9 months) and the TTSP was 358 days (11.7 months).

**Figure 2:**
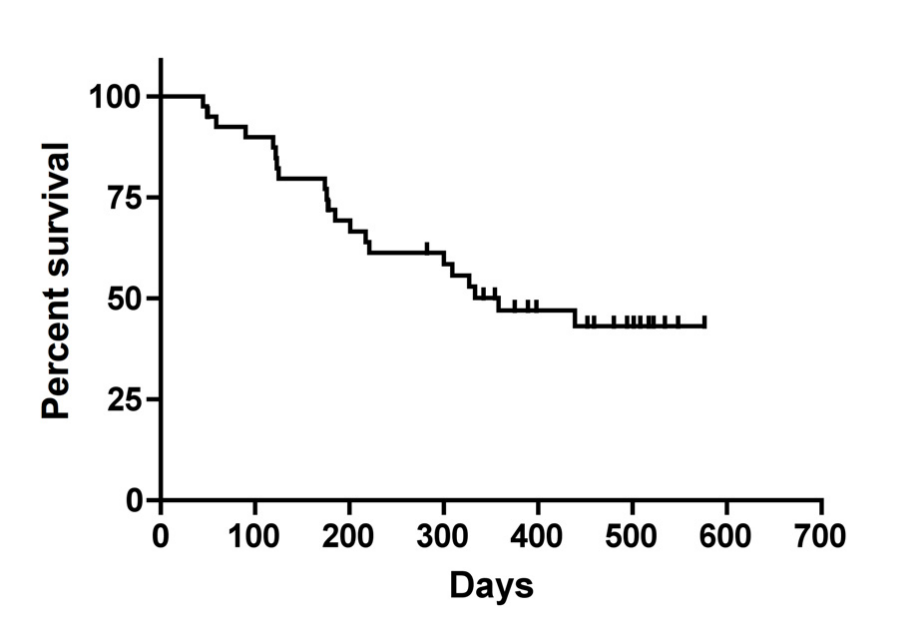
**Kaplan-Meier Analysis of Overall Survival and Progression Free Survival**. Among 40 patients, the median overall survival was 358 days (11.7 months) and the progression free survival was 57 days (1.9 months).

No complete or partial responses were observed in patients treated with low dose cyclophosphamide and GV1001. Stable disease was observed in 17 patients (45.9%) as the best response during follow-up. Twenty patients demonstrated a progressive disease and three patients were not assessed for tumor response after screening due to clinical progression or death before treatment was initiated. The majority of patients (35 patients, 87.5%) had tumor progression by the end of the study. 5 patients were lost to follow-up. The median TTP for the patients was 57.0 days (95% CI 52.0 - 102) as shown in Figure 1. The evaluation of TTSP showed that a total of 21 patients (52.5%) in the ITT population had symptomatic progression (a 2-step deterioration in performance status) or died prior to the end of the study, and 19 patients (47.5%) were censored at the date of their last visit or contact as they had no documented symptomatic progression at the end of the study. The median TTSP was estimated to be 358.0 days (95% CI: 217.0; -) (Figure 1). A total of 36 patients (90%) in the ITT population had tumor progression or death from any cause prior to the end of the study. The median PFS was 57.0 days (95% CI 52.0 - 96.0) (Figure 2). Finally, overall survival was analyzed in the patient population. The estimated median OS for the ITT population was 358.0 days (95% CI 217; upper limit could not be calculated) (Figure 2).

### Immune response analysis

**Figure 3:**
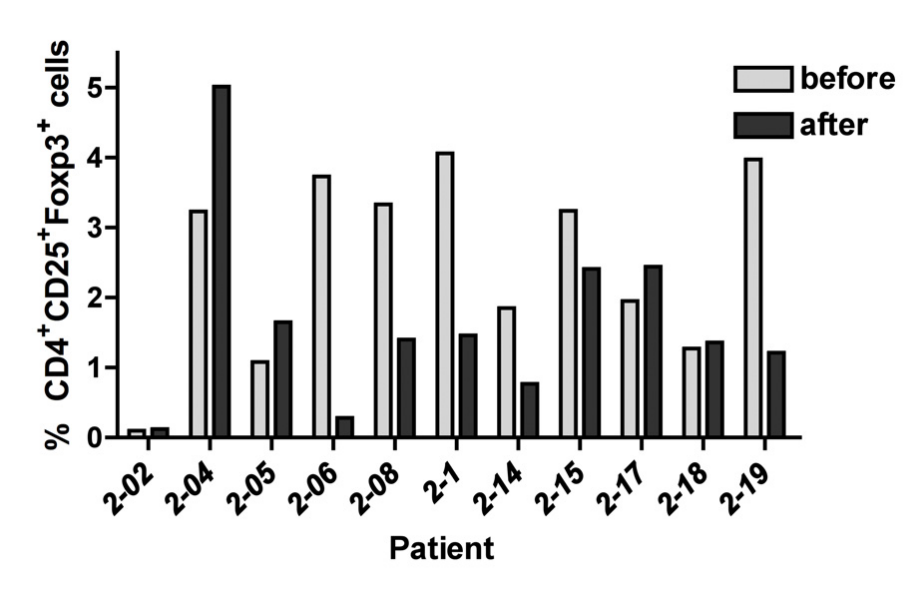
**Analysis of CD4^+^CD25^+^Foxp3^+ ^regulatory T cells in HCC patients prior to cyclophosphamide treatment and after 5 days**.

**Figure 4:**
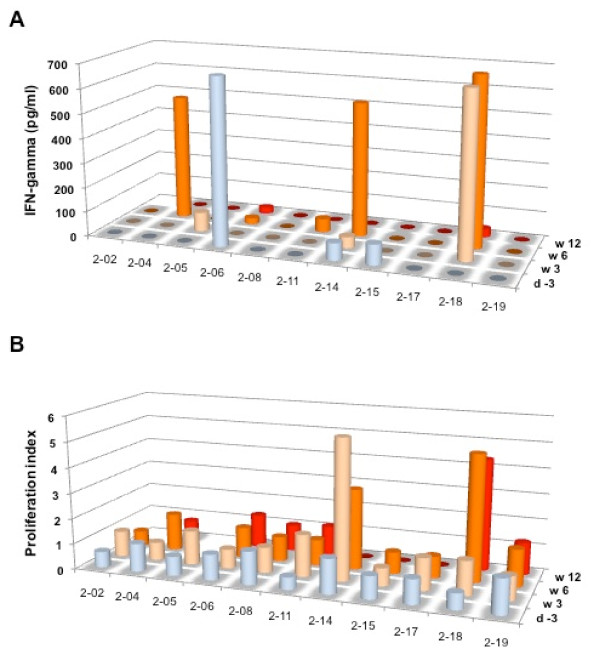
**Analysis of GV1001 specific T cell responses by ELISA (A) and thymidine incorporation (B) in HCC patients before and after peptide vaccination**. Among 11 patients analyzed no definite antigen-specific T cell responses were noticed.

Three patients responded to the DTH test. However, two of these patients already demonstrated a DTH response prior to immunization. One patient demonstrated a DTH response 2 weeks after vaccination. However, this DTH response was not observed at any of the later time points. T-cell responses have only been carried out for patients treated at one of the three sites (Germany) for logistic reasons. The frequency of CD4^+^CD25^+^Foxp3^+ ^regulatory T cells was determined by FACS analysis before and five days after cyclophosphamide treatment. A decrease in the relative frequency of CD4^+^CD25^+^Foxp3^+ ^regulatory T cells was found in 6/11 patients (54.5%) (Figure 3). GV1001 specific T cell responses were analyzed by cytokine secretion as well as proliferation analysis. However no clear GV1001 specific T cell responses were observed in any of the patients after pretreatment with cyclophosphamide and immunization with GM-CSF and GV1001 peptide (Figure 4).

## Discussion and conclusion

Until recently, no systemic treatment has shown any benefits for patients with advanced HCC. The breakthrough results obtained with sorafenib have represented a major step in the management of this deadly disease that now has an option of similar efficacy as that of agents used for other cancers such as lung and colorectal [15]. Unfortunately the impact of sorafenib is not the final answer. Tumor growth is significantly delayed and survival is improved, but ultimately the HCC progresses and patients die because of cancer. This shows the need to develop new agents that would further expand the benefits of current therapy [16]. Major interest is being placed in the evaluation of other molecular therapies to be given in association to sorafenib or upon sorafenib failure, thus opening the field for second line options. In addition to agents modulating the activation of signaling cascades, there is a known potential in priming immune response against cancer cells to overcome the immune escape that malignant cells are able to establish as a prominent feature [5]. Several approaches to induce immune reactivation to control cancer have been tested [17]. In this investigation, we have evaluated the effect of a telomerase derived peptide vaccine in combination with low dose cyclophosphamide treatment in a single arm phase II trial. The primary efficacy variable for this study was overall response; however, there were no complete or partial responses during this study. The best overall tumor response (according to RECIST) for the ITT population was stable disease for 17 patients (45.9%). In a recent placebo-controlled phase III study on Sorafenib in advanced HCC patients (SHARP trial), none of the patients had a complete response, but 2% of the patients in the Sorafenib group had a partial response and 71% had stable disease (according to RECIST) maintained for at least 28 days after the first demonstration [3]. However, in the Sorafenib study, tumor measurements were performed every 6 weeks whereas in the present study CT scans were performed every 8 weeks, which makes direct comparison of tumor response between studies difficult. Nonetheless, the TTP detected in our study with a less frequent timing is shorter than that registered both in the SHARP and in the Asian Pacific trials. Hence, vaccine therapy using GV1001 has not evidenced any efficacy in terms of tumor response and TTP. This has been recently suggested to be the optimal end-point to register the potential efficacy of agents where no major reduction in tumor mass is to be expected [2]. The validity of the concept has been shown in the sorafenib studies and currently, most investigations are designed to capture at the same time TTP and tumor response according to conventional RECIST with the modifications reflected in the JNCI AASLD guidelines [2].

In the present study, the majority of patients (35 patients; 87.5%) in the ITT population had disease progression by the end of the study with a median TTP of 57.0 days (95% CI: 52.0; 102.0). Median PFS was also 57.0 days (95% CI: 52.0; 96.0). In the Sorafenib study, the median time to radiologic progression was 5.5 months in the Sorafenib group and 2.8 months in the placebo group [3]. It should be noted, however, that the baseline characteristics of the patients in the Sorafenib study were different to the baseline characteristics of the patients in the present study. Only patients with an ECOG performance status 0 or 1 were enrolled in the present study, whereas 7-8% of patients in the Sorafenib study had an ECOG performance status 2 at baseline. Furthermore, more patients in the present study (77.5%) had an ECOG performance status 0 at baseline compared with patients in the Sorafenib study (54%). Similarly, more patients in the present study had a BCLC stage of A or B (35%) at baseline compared with patients in the Sorafenib study (18%). Hence, patients in the present study tended to have less advanced disease at baseline compared with the baseline status of patients in the Sorafenib study. Despite this, the median TTP was shorter in patients in the present study than in patients in the placebo group of the Sorafenib study. However, median OS was 358.0 days (95% CI: 217.0; upper limit not calculable) (11.5 months) in comparison to 10.7 months in the Sorafenib group and 7.9 months in the placebo group of the SHARP trial [3]. The longer median OS in patients treated with GV1001 compared with the median OS in both the placebo and patients in the SHARP trial could be explained by the better baseline condition of the patients in the present study. This is why overall survival should not be an informative endpoint in phase 1-2 studies as the bias in the selection of patients for an experimental intervention sure can induce a survival that might be misleadingly. It could be argued that vaccination could take some time to be effective and hence delay late tumor progression, while at early follow-up time points, the benefit would not be captured. Such an evaluation would require a different study design and development of assessment criteria that are not available and validated. In addition, after detecting tumor progression within this investigation, patients may have engaged in other experimental approaches and thus, it is unfeasible to explore this later evolutionary profile.

In contrast to previous studies in which patients with pancreatic cancer or non-small lung cancer were immunized with GV1001 [9,10], no clear GV1001 specific immune responses were observed in HCC patients after treatment with low dose cyclophosphamide followed by repetitive GM-CSF/GV1001 immunizations. We cannot exclude that the pre-treatment administration of a single dose of 300 mg/m^2 ^cyclophosphamide in this trial with the purpose of overcoming the effects of inhibitory effects by regulatory T cells may have influenced the immune responses in the DTH test as well as our ex vivo T cell analysis. However, based on the results from our previous trial, in which we treated advanced HCC patients only with cyclophosphamide [13], we did not expect any effects on antigen-specific T cell responses since in this trial cyclophosphamide treatment had no significant effect on the frequency of CD4^+ ^or CD8^+ ^T cells. In contrast, we were able to detect spontaneous tumor-specific immune responses after cyclophosphamide treatment in a limited number of patients. Moreover, low dose cyclophosphamide treatment has also been used in a number of other clinical trials, where it potentially supported the effect of different vaccines [18,19] and no effect on antigen-specific immune responses were observed in a number of different preclinical studies in mice [20-22].

Cyclophosphamide, GV1001 and GM-CSF treatment were in general well tolerated in this study. There were no adverse events > CTC 2 for GV1001 or GM-CSF treatment and the majority of the observed adverse events were related to the injection procedure and injection site reactions. One Grade 3 adverse event (renal failure) was observed in a patient treated with cyclophosphamide, which was reversible. Therefore the treatment was much less toxic than other treatments such as sorafenib [3] or other molecular targeting agents [16].

In summary, our study failed to demonstrate significant tumor responses. This might be due to the fact, that in contrast to other GV1001 immunization trials, no clear immune responses (DTH or T cell responses) have been observed in this study. One possibility is the addition of cyclophosphamide in order to target regulatory T cells or the nature of the disease, although clear T cell responses have been observed in other vaccination trials [23-25]. Further studies are needed to analyze the effect of a combined chemo-immunotherapy, which will be interesting in light of recent data, which suggest that Sorafenib, which has become the standard of care for patients with advanced HCC has significant effects on tumor-specific immune responses [26,27].

## Competing interests

This study was supported by Pharmexa A/S Biosciences to TFG, MB and JB.

## Authors' contributions

TFG, MB and JB were critically involved in the study design, statistical analysis. TFG, AF, GK, NB, CA, MPM, MB and JB recruited patients for this trial. TFG, FK and LAO were involved in the analysis of regulatory T cells. TFG drafted the manuscript, which was revised by FK, MB and JB. All authors have read and approved the final manuscript.

## Pre-publication history

The pre-publication history for this paper can be accessed here:

http://www.biomedcentral.com/1471-2407/10/209/prepub
